# Toward Synthesizing Our Knowledge of Morphology: Using Ontologies and Machine Reasoning to Extract Presence/Absence Evolutionary Phenotypes across Studies

**DOI:** 10.1093/sysbio/syv031

**Published:** 2015-05-26

**Authors:** T. Alexander Dececchi, James P. Balhoff, Hilmar Lapp, Paula M. Mabee

**Affiliations:** ^1^Department of Biology, University of South Dakota, Vermillion, SD 57069, USA;; ^2^National Evolutionary Synthesis Center, Durham, NC 27705, USA;; ^3^University of North Carolina, Chapel Hill, NC 27599, USA;; ^4^Center for Genomics and Computational Biology, Duke University, Durham, NC 27708, USA

**Keywords:** character conflict, evolutionary mapping, inference, missing data, morphological character, ontology, phenotype, supermatrix

## Abstract

The reality of larger and larger molecular databases and the need to integrate data scalably have presented a major challenge for the use of phenotypic data. Morphology is currently primarily described in discrete publications, entrenched in noncomputer readable text, and requires enormous investments of time and resources to integrate across large numbers of taxa and studies. Here we present a new methodology, using ontology-based reasoning systems working with the Phenoscape Knowledgebase (KB; kb.phenoscape.org), to automatically integrate large amounts of evolutionary character state descriptions into a synthetic character matrix of neomorphic (presence/absence) data. Using the KB, which includes more than 55 studies of sarcopterygian taxa, we generated a synthetic supermatrix of 639 variable characters scored for 1051 taxa, resulting in over 145,000 populated cells. Of these characters, over 76% were made variable through the addition of inferred presence/absence states derived by machine reasoning over the formal semantics of the source ontologies. Inferred data reduced the missing data in the variable character-subset from 98.5% to 78.2%. Machine reasoning also enables the isolation of conflicts in the data, that is, cells where both presence and absence are indicated; reports regarding conflicting data provenance can be generated automatically. Further, reasoning enables quantification and new visualizations of the data, here for example, allowing identification of character space that has been undersampled across the fin-to-limb transition. The approach and methods demonstrated here to compute synthetic presence/absence supermatrices are applicable to any taxonomic and phenotypic slice across the tree of life, providing the data are semantically annotated. Because such data can also be linked to model organism genetics through computational scoring of phenotypic similarity, they open a rich set of future research questions into phenotype-to-genome relationships.

The analysis of phenotypic traits in a phylogenetic framework is key to addressing the evolutionary questions posed by an increasingly diverse set of domains. For example, understanding the evolution of pharyngeal jaw mechanics in fishes (Price et al. 2010), identifying phenotype-associated genes and regulators in forward genomics approaches (Hiller et al. 2012), exploring the key factors in land plant evolution (Rudall et al. 2013), or discovering the role of phenotypic traits in colonization ability (Van Bocxlaer et al. 2010), all rely on the mapping of phenotypic data to phylogeny. Although robust molecular phylogenies have become easier to generate, more broadly available, and increasingly comprehensive, the phenotypic data on which these studies rely have not.

Unlike molecular data, phenotypic data are notoriously time-consuming and complex to observe, classify, and code (Burleigh et al. 2013). Moreover, they are described in a highly detailed free-text format in a distributed literature and have not been available in a computable format (Deans et al. 2012). Researchers seeking to aggregate even the seemingly simple information about the presence and absence of phenotypes across a set of species are faced with a substantial manual extraction and abstraction task (e.g., Stewart et al. 2014). Although assertions of the presence and absence of phenotypes abound in the literature, so do descriptions of the variation in other qualities such as shape, size, position, color, etc. In the case of these qualities, presence and absence must be inferred; from the description “posterior flap of adipose fin, free from back and caudal fin” (Lundberg 1992), the adipose fin would be assumed present. Such detailed data, originally collected for phylogenetic reconstruction or taxonomic identification, are desirable for re-use at the more general level of presence and absence where they pertain to broader questions concerning, for example, homoplasy, rates, and correlations of phenotype with environment, geography, and genes.

Here we show that the presence and absence of phenotypes can be extracted automatically from published detailed phenotype descriptions that are annotated using ontologies. For example, if an author asserts that a fin ray is branched in a particular fish species, we can use the logic inherent in the corresponding ontology-based expression to infer that the fin ray is present. The power of inference across ontology-based phenotypes (Balhoff et al. 2010; Dahdul et al. 2010a; Mabee et al. 2012) from multiple species and multiple studies enables a substantial reduction in the proportion of missing data in a matrix. We here demonstrate that the logical inferences enabled by ontologies significantly expand the coverage of the data, revealing gaps in phenotype and taxon sampling, and revealing data conflict across studies. The methods described here not only allow aggregation of phenotypic data into synthetic supermatrices, but also show the need to more broadly adopt the use of ontology annotation in the morphological literature to facilitate linking and integration with other data such as genetic and developmental data from model organisms (Mabee et al. 2012).

## Methods

The methods described here rely on the use of ontologies. Because the application of ontologies to systematics is recent, we provide a glossary to aid the reader (Box 1).

Box 1. Glossary of key terms used in the description of anatomy data synthesis

**Annotation**—The application of one or more ontology terms to a text expression such as a character state.

**Assertion**—A direct author statement about a fact or observation, which here is usually an organismal phenotype, typically deduced from observations about a specimen, including whether an entity is present or absent in an organism.

**Character state**—One of a subset of pre-defined values used in the composition of characters to differentiate between taxa. Character states are published in natural language text that is not computable.

**Entity**—An entity is a feature of an organism, such an anatomical element, a molecular process, or a behavior. It is represented in an ontology by a class, for example, an anatomy ontology class such as “pectoral fin.”

**EQ**—Entity–Quality, the combination of an entity class with a quality class. An EQ is an ontology-based representation of a phenotype (e.g., pectoral fin, presence).

**Inference**—A statement about a fact, such as the presence or absence of an entity, that is implied by one or more asserted facts by means of the logical relations of an ontology.

**Inheres in**—The relation between a dependent continuant (in this case a quality) and an entity. For example, the triangular shape that inheres in the deltopectoral crest of a tyrannosaur.

**Ontology**—Ontologies are hierarchical vocabularies with well-defined relationships between terms that can be used by computers to integrate data.

**OWL**—Web Ontology Language, a language standardized by the World Wide Web Consortium (WC3) for defining first-order logic ontologies.

**Phenotype**—A feature of an organism and its quality. May be represented with ontologies using Entity–Quality (EQ) syntax.

**Quality**—A quality describes the particular way that an entity varies in presence, size, shape, composition, etc. It is represented in an ontology (a quality ontology like PATO) by a class, for example, a quality ontology class such as “present” or “curved.”

**Reasoning**—The use of logic, here embodied in an ontology, to reach a conclusion. Inference is a type of reasoning.

**Subclass**—A derived class that inherits properties from its parent classes, and usually has some of its own. For example, a “radius bone” is a subclass of “bone” and thus inherits its properties, such as being composed of bone tissue.

**Synthetic character**—A character whose states are assembled from multiple studies using the logical relationships specified in an ontology.

**Synthetic supermatrix**— A character matrix consisting of synthetic characters.

**Figure 1.**
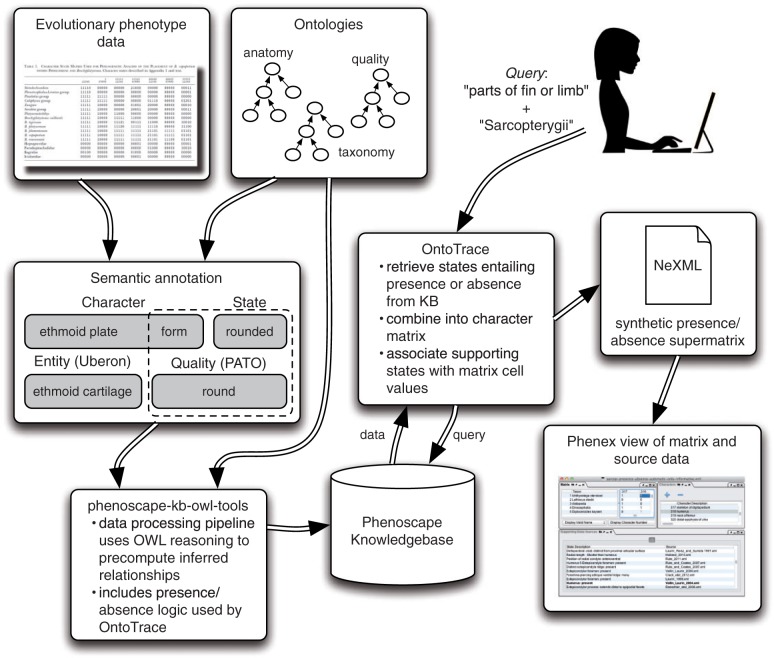
Flow chart showing computational steps used to extract synthetic presence/absence supermatrices from ontology-annotated evolutionary phenotype data. Phenotypic character states of taxa from the evolutionary literature are semantically annotated using anatomy, quality, and taxon ontologies. Using the phenoscape-kb-owl-tools data processing pipeline (https://github.com/phenoscape/phenoscape-owl-tools), these phenotypes are reasoned across and deposited into the Phenoscape Knowledgebase. The OntoTrace tool enables a user to generate synthetic presence/absence matrices for specific taxa (here “Sarcopterygii”) and particular anatomical entities (here “parts of fin or limb”). These matrices, including provenance for each cell, can be viewed in Phenex.

The Phenoscape Knowledgebase (KB; kb.phenoscape.org) contains ontology-annotated phenotype data derived from published character state matrices from phylogenetic treatments (Fig. 1). Annotations are ontological expressions composed according to the Entity–Quality (EQ) formalism (Mungall et al. 2007, 2010), using the Phenex software (Balhoff et al. 2010) as described previously (Dahdul et al. 2010a) (Fig. 1). Anatomical entities are represented by terms from the comprehensive Uberon anatomy ontology for metazoan animals (Mungall et al. 2012; Haendel et al. 2014), which was derived in part from independently developed vertebrate multispecies ontologies (Dahdul et al. 2010b; 2012; Maglia et al. 2007). The Uberon ontology includes explicit, expert community-vetted, and sourced statements about homology, and splits anatomical structures named homonymously between clades but known to be nonhomologous into separate classes for each clade (see Haendel et al. 2014). For example, the tetrapod parietal bone and the actinopterygian parietal bone form distinct classes in the ontology (and each is a subtype of neurocranium bone), preventing inference methods from inadvertently treating structures as the “same” that are known not to be. Phenotypic qualities (presence/absence, size, shape, composition, color, etc.) are drawn from the Phenotype and Trait Ontology (PATO) (Gkoutos et al. 2005). Terms for vertebrate taxa are taken from the Vertebrate Taxonomy Ontology (VTO) (Midford et al. 2013). Every morphological matrix annotated in this way is associated with a single publication in the KB.

At the time of this analysis, the KB (Fig. 1) contained a total of 19,024 morphological character states corresponding to 651,660 EQ phenotype annotations for 4399 extant and fossil vertebrates from 139 comparative studies. It is particularly enriched in the comparative skeletal anatomy for fins, limbs, and their support structures (girdles) of sarcopterygian vertebrates (Table 1 available as Supplementary Materials on Dryad at http://dx.doi.org/10.5061/dryad.rm907), the clade in which the “fin-to-limb” transition occurred (see Shubin et al. 2014 for a recent discussion). Sarcopterygii comprise slightly greater than half of all vertebrates (Barnosky et al. 2011) and include lobe-finned fishes such as lungfish and coelacanths, and tetrapods including amphibians and amniotes. These richly annotated taxa and phenotypes served as the source data for this investigation.

To automate synthesis of supermatrices from the phenotype-by-taxon knowledge in the KB, we created the OntoTrace tool (Fig. 1). OntoTrace accepts as input (i) the targeted anatomical elements (or regions) in the form of a pertinent ontology class or expression (specifically, an OWL class expression), and (ii) the taxonomic group(s) (also in the form of an OWL class expression) for which a supermatrix is to be synthesized. OntoTrace first generates a matrix column, and thus a character, for each anatomy ontology class subsumed by the input class expression. Then, for each anatomical character so generated, OntoTrace queries the KB for character states whose EQ annotations logically entail the presence or absence of the respective anatomical element, given the subclass relationships, part–whole relationships, developmental origin, and other axioms provided by the requisite ontologies (see below). The taxa that are associated with those character states and that fall within the input taxonomic group (i.e., are subsumed by the input class expression designating the taxa of interest) are then added to the matrix as rows, and they are given a state of present, absent, or both (as a polymorphism) for the character, as entailed by their respective character states (more precisely, by the EQ annotations for those states). To document the provenance of each synthetic state, all combinations of taxon and published character state supporting the synthetic state value(s), along with references to the respective source matrices (and thus publications), are recorded as metadata for each cell in the synthetic matrix. In addition, OntoTrace determines whether any of the published states supporting a synthetic state directly assert, in the form of their EQ annotations, the presence or absence of the anatomical element, or whether the synthetic state is solely based on logical inference from the supporting states' EQ annotations. Direct assertion of presence/absence here means that the curated EQ annotation(s) for the respective state uses the respective character's anatomical structure as the entity (E), and one of the terms “present” (PATO:0000467) or “absent” (PATO:0000462) as the quality (Q). OntoTrace outputs the generated matrix and all metadata in a single file in the NeXML format (Vos et al. 2012) (Fig. 1). OntoTrace is implemented in the Scala programming language, and its source code is freely available under the MIT license on GitHub at https://github.com/phenoscape/ontotrace. The source code also contains several ancillary reporting scripts we used to review properties of the matrix (described below). The version of OntoTrace described here has been archived at http://dx.doi.org/10.5281/zenodo.12705.

**Figure 2.**
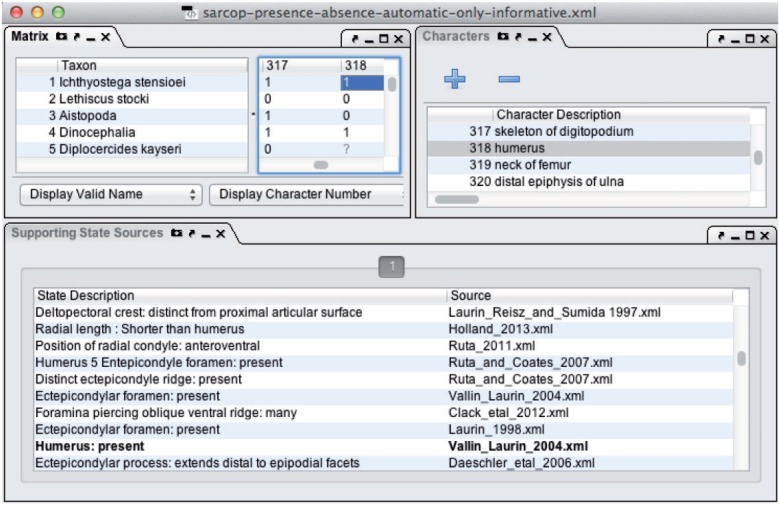
Screenshot from Phenex, showing a portion of synthetic supermatrix in Matrix panel (left), synthetic characters in Characters panel (right), and provenance in the new Supporting State Sources panel (below). Here the Supporting State Sources panel displays the sources of the character states for the synthetic character 318 “humerus” in *Ichthyostega stensioei*.

To allow manual review of the provenance of the cells in the generated synthetic presence/absence supermatrices, we developed a new interface panel for Phenex, the EQ annotation tool (Balhoff et al. 2010) (Figs. 1 and 2). Upon selection of a matrix cell in Phenex, the new Supporting State Sources panel displays the list of originally published character states that support the presence/absence state value assignment(s) for the respective taxon, and Phenex highlights in bold those that are considered supporting by direct assertion rather than by inference (Fig. 2).

To illustrate the properties and value of synthetic morphological supermatrices, we aimed to generate a synthetic presence/absence supermatrix of any anatomical elements that are part of the paired limb, paired fin, and/or the girdle skeletons for any sarcopterygian taxa. We also included elements that are connected to these structures, such as the sternum. To achieve this, we selected anatomical structures using the following OWL class expression, shown below with term labels rather than identifiers for readability:

*part_of* some (‘paired limb/fin’ or ‘girdle skeleton’) or *connected_to* some (‘paired limb/fin’ or ‘girdle skeleton’)

The properties *part_of* (BFO:0000050) and *connected_to* (RO:0002170) are from the OBO Relations Ontology (Smith et al. 2005), and the classes “paired limb/fin” (UBERON:0004708) and “girdle skeleton” (UBERON:0010719) are from the Uberon anatomy ontology (Mungall et al. 2012; Haendel et al. 2014). The taxa were selected using the VTO (Midford et al. 2013) term “Sarcopterygii” (VTO:001464) as input, which permitted us to generate data for taxa annotated to Sarcopterygii or any of its subclasses in VTO. We ran OntoTrace on a Linux-based compute node, using 60 GB RAM, within Duke University's shared high-performance computing cluster, with a build of the Phenoscape KB generated on 23 June 2014.

### Entailment of Presence and Absence

**Figure 3.**
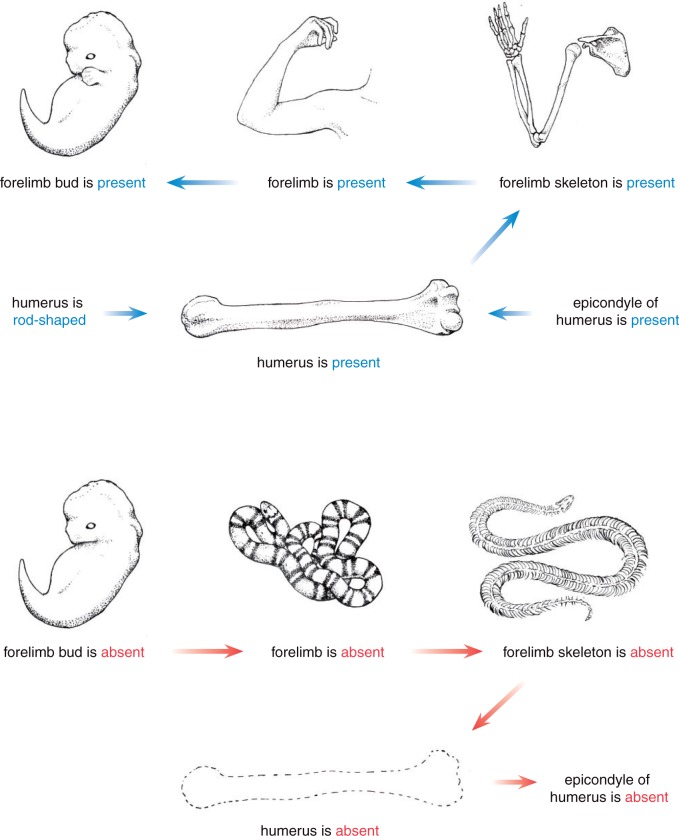
Ontology-based inference of presence and absence. Direction of arrows indicate the reasoning pathway. Top: the presence of a structure (humerus) is inferred from an assertion to its shape (humerus L-shaped) or a part (entepicondyle of humerus is present). The presence of humerus implies the presence of forelimb skeleton (humerus is part of a forelimb skeleton), a forelimb (forelimb skeleton is part of a forelimb), and thus a forelimb bud (forelimb develops from a forelimb bud). Bottom: in contrast, an assertion to the absence of a humerus does not entail the absence of a forelimb bud, forelimb, or forelimb skeleton; it does entail the absence of its parts (entepicondyle). However, the absence of a forelimb bud entails the absence of a forelimb, thus a forelimb skeleton and thus the humerus.

The ontologies from which we draw our terms provide a rich context with a community-vetted set of definitions and relationships (structural, developmental) for each entity. The semantics of the OWL ontology language used by Phenoscape, Uberon, and the major model organism ontology communities permits a rich set of inferences to be derived from EQ annotations either in a developmental phenotypic context (as used by model organism databases) or, as seen here (Fig. 3), in an evolutionary phenotypic context. For example, a simple EQ annotation may assert that a character state describes an entity “humerus” bearing a quality “L-shaped.” A state assignment to a taxon implies that the taxon has a member organism that exhibits a phenotype, that is, has an instance of the ontology class “L-shaped” that inheres in an instance of the class “humerus.” In the EQ model (Mungall et al. 2007, 2010), the relation inheres_in (RO:0000052) from the OBO Relations Ontology (Smith et al. 2005) connects phenotypic qualities to the anatomical entities that bear them. Based on the assertion that there is an organism exhibiting a phenotypic quality inhering in a structure, we can trivially infer that this structure must be present in the organism. Using an OWL reasoner and additional axioms provided by the Phenoscape KB, more indirect inferences of presence or absence can be made as well, which essentially result from the anatomical knowledge expressed within the Uberon ontology (Balhoff et al. 2014).

#### Presence

To query character states that denote presence of a given structure, OntoTrace retrieves phenotypes from the KB that are subsumed by the expression “*implies_presence_of* some <entity>.” *Implies_presence_of* is an OWL property that unifies the various means by which the presence of a structure can be inferred (see Balhoff et al. 2014 for details). For example, a quality that *inheres_in* a structure *implies_presence_of* that structure (Fig. 3). Presence is also inferred for any structures of which that structure is a part or from which it develops. The presence of a “humerus” implies the presence of a “forelimb” and a “forelimb skeleton,” of which Uberon asserts it to be a part. The presence of a “forelimb” also implies the presence of a “forelimb bud,” because Uberon asserts that the former develops from the latter (Fig. 3).

#### Absence

To query character states that denote absence of a given structure, OntoTrace retrieves from the KB those phenotypes that are subsumed by the expression “lacks_all_parts_of_type and *inheres_in* some multicellular_organism and *towards* value <entity>.” Similar to “presence,” the KB makes use of chains of ontological relationships to infer which other structures must be absent as the consequence of the absence of a given structure (Fig. 3) (see Balhoff et al. 2014 for details). For example, the absence of a “forelimb” entails the absence of a “humerus.”

### Identifying Conflicts

When a taxon is inferred to exhibit both presence and absence for a particular structure, it indicates either a polymorphic condition in the taxon, or the fact that the supporting original character states, or more precisely, the EQ annotations made for them, conflict with each other. Polymorphic synthetic state values were considered as reflecting actual polymorphism, and thus excluded from further review, if both presence and absence are directly asserted by supporting character states associated with a single source matrix (and thus the same publication). To aid manual review of the remaining conflicts, we created a script that reported for each conflict the taxon, the entity that was polymorphic (i.e., had conflicting values), and whether the presence and absence values were supported by direct assertion or inference. This reporting script can be found in the OntoTrace source code repository. Provenance of conflicting data can be viewed in Phenex (Fig. 2).

### Identifying Isomorphic Synthetic Characters

To examine possible dependence across characters in the synthetic matrix as a consequence of assertions in the ontologies, we used a script to report each cluster of characters (i.e., anatomical entities) that were identical in their taxonomic distribution of values. In other words, all identical character columns were collected into clusters; we term these clusters “isomorphic characters.” Further, for each of the anatomical entities comprising each cluster, the script reported whether the matrix contained any direct presence/absence assertions for that character, or if it was included in the matrix solely through inference. Code for this report can be found in the OntoTrace source repository. To aid in characterization of the ontological dependence of isomorphic characters, we used a script to generate an ontology of “presence classes”: For each anatomical entity “X” from the Uberon ontology, we generated a corresponding class with the logical definition “*implies_presence_of* some X.” We classified these expressions using the ELK reasoner (Kazakov et al. 2012, 2013) within the Protégé ontology editor, and used the Protégé DL Query panel to check for inferred equivalency between presence expressions.

### Other Reporting Queries

The number of published character states that entail the presence or the absence for selected sets of entities and taxa was reported using queries to the Phenoscape KB implemented as a script included within the OntoTrace source code. Specifically, for each taxon and entity, we queried for states that were annotated with phenotypes that entailed either the presence or the absence of the entity.

We queried (using SPARQL, the query language for RDF datastores) the Phenoscape KB to count the number of published matrices in which each sarcopterygian taxon in the KB is included. An additional query was used to report, for each published matrix in the KB, the number of taxa, characters, states, and phenotypes associated with annotations relevant to structures of the fin or limb. These queries are included in SPARQL format within the OntoTrace code repository.

## Results

OntoTrace aggregated, as described above, the KB's morphological phenotype data on paired limb, paired fin, and/or the girdle skeletons for all sarcopterygian taxa into an entity-by-taxon matrix of 1759 synthetic presence/absence characters by 1052 taxa, in the form of an XML file in NeXML format (Vos et al. 2012) made available in the Dryad data repository (see sarcop-presence-absence-all.xml in Supplementary Materials on Dryad at http://dx.doi.org/10.5061/dryad.rm907). The 55 studies from which data were synthesized in this manner are summarized in Table 1 available as Supplementary Materials on Dryad at http://dx.doi.org/10.5061/dryad.rm907. The data from these papers that relate to fin, limb, girdle and their parts total 2588 text-based character states from 1195 individual published characters, scorable for 1052 sarcopterygian taxa.

**Figure 4.**
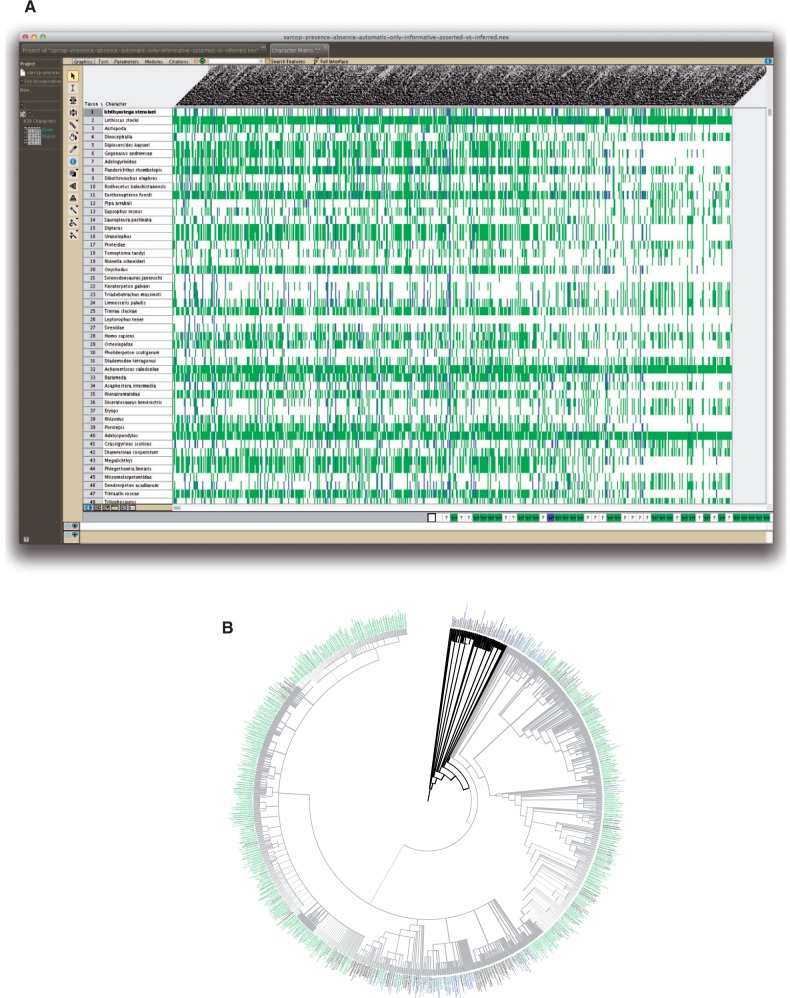
A) Bird's Eye View in Mesquite (Maddison and Maddison 2014) showing inferred (green), asserted (blue), and missing (white) data in the synthetic supermatrix for the first 48 taxa (of 1051) and all 639 characters. B) Phylogeny of sarcopterygian vertebrates (Tetrapoda in grey) represented in the synthetic supermatrix, showing the distribution of data for one character, “skeleton of digitopodium.” Tip labels in black denote the absence of data, blue denotes taxa with asserted data, and green denotes taxa with inferred data.

Out of the 1759 generated synthetic characters, 639 were variable, that is, included both presence and absence states. Of these, 488 characters (76%) were variable only due to the use of inference: 442 variable characters were composed of inferred data alone; 12 were made variable by inferred absence, and 34 by inferred presence. In the matrix subset comprising the variable characters there are 146,451 populated cells, which constitute 21.8% of all cells. Directly asserted data accounted for only 9948 (6.8%) of the populated cells, or 1.5 % of all cells in the subset; in contrast, inferred data represent 93.2% of the populated cells (Fig. 4). Of the 1051 taxa in the subset, 13% (136 taxa, see Table 2 available as Supplementary Materials on Dryad at http://dx.doi.org/10.5061/dryad.rm907) are included in the matrix solely on the basis of inferred data. Taxa for which the source matrices contain no direct assertions about presence or absence of any fin/limb entity can nonetheless be included in the synthetic presence/absence matrix if they have EQ phenotype annotations that imply presence or absence of a fin/limb entity. For example, the theropod dinosaur taxon *Sinosauropteryx prima* in our data is derived from a single source (Sereno 2009), where it was not scored for any presence/absence characters. Its inclusion in the synthetic supermatrix comes solely from character states such as “increased scapular blade width” and “poorly differentiated humeral head form,” because these imply the presence of a scapula and humerus, respectively.

After excluding polymorphisms directly asserted within a single source (see “Identifying conflicts” section), we identified 774 cells (of the 146,451 populated ones) as stating both presence and absence (0/1) of a character, for 99 synthetic characters and 297 taxa. These included 135 conflicts between direct assertions (that were made in different source publications), 565 conflicts between direct assertions and inferred states, and 74 conflicts between inferred states (Table 3 available as Supplementary Materials on Dryad at http://dx.doi.org/10.5061/dryad.rm907).

We also identified 93 clusters of characters in the synthetic supermatrix that were isomorphic, that is, identical in their distribution across taxa and to one another, but variable. These correspond to 85,813 cells in the synthetic supermatrix (Table 4 available as Supplementary Materials on Dryad at http://dx.doi.org/10.5061/dryad.rm907), almost 59% of the (populated) cells. To better characterize these clusters as to their ontological basis, we examined which of them fall into equivalence chains of implied presence and absence. More specifically, two synthetic characters with anatomical entities X and Y, respectively, for which a reasoner infers equivalence between the logical definitions “*implies_presence_of* some X” and “*implies_presence_of* some Y” will necessarily be found isomorphic in their distribution of presence and absence. For example, “presence of pedal digit 2” is inferred as equivalent to “presence of pedal digit 2 digitopodial skeleton.” Twenty-one of the 93 clusters (23%) were of this kind. Another 6 (6%) were found to be clusters of anatomical parts and the entities that contain them. Clusters can also arise from co-asserted entities (e.g., when a single character state includes multiple entities, such as “pedal digits 6, 7, and 8 present,” which will result in three EQ annotations, one for each of the three digits). There were three such clusters (3%). Most of the clusters were composed of inferred data only. Of these, 63 (68%) resulted from chains of inference from multiple entities that were different for each cluster.

The number of source matrices from which a particular taxon was sampled ranged from 1 to 16 (Table 5 available as Supplementary Materials on Dryad at http://dx.doi.org/10.5061/dryad.rm907). In all, 813 (77.4%) of the sampled taxa are at the species rank, with the remainder distributed across higher-level ranks (Table 5).

**Figure 5.**
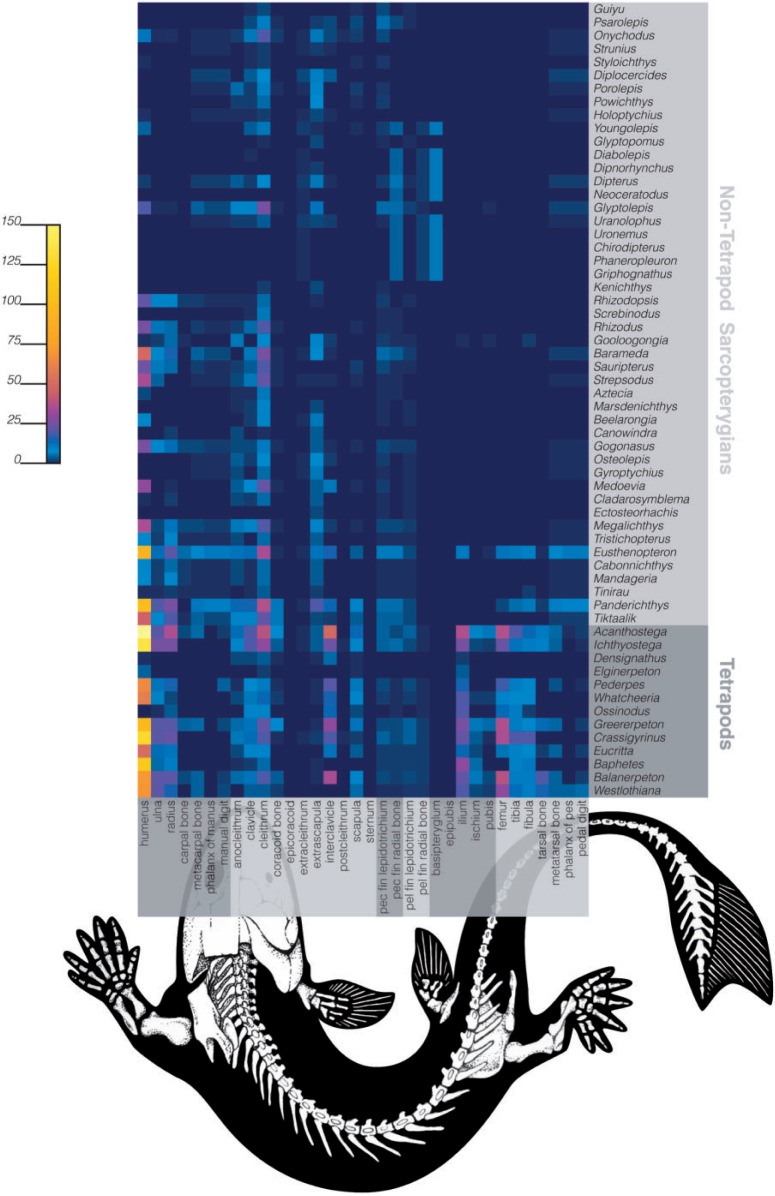
The level of anatomical data available for different parts of the fin and limb can be visualized for taxa along the fin-to-limb transition. Taxa included in this analysis encompass all major clades from the base of Sarcopterygii to the basal amphibians *Baphetes* and *Westlothiana* (see Ruta 2011 and Clack et al. 2012 for source topology). All taxa in this analysis are extinct with exception of the lungfish *Neoceratodus*. Taxa lacking all data for fin or limb were excluded. Cell color reflects the number of character states that entail the presence or the absence of that entity for each taxon (row).

The number of published character states that entail the presence or absence for selected parts of the fin and limb was used to generate a figure showing their distribution across taxa along the fin-to-limb transition (Fig. 5, Table 6 available as Supplementary Materials on Dryad at http://dx.doi.org/10.5061/dryad.rm907).

## Discussion

The first step in scaling up the exploration of phenotypic patterns in an evolutionary context is to render phenotypic descriptions of species in a form amenable to large-scale computational integration, linking, and mining. How this is possible has recently been shown in a series of papers from the Phenoscape group (Dahdul et al. 2010a; Mabee et al. 2012). Here we demonstrate that, using computable phenotypes from a datastore representing the cumulative effort of experts across a broad taxonomic scale, synthetic supermatrices for presence/absence phenotypes can be automatically assembled for user-designated slices of the taxonomic and anatomical corpus.

Bringing together phenotypic data from across multiple studies manually, and synthesizing them in a form amenable to computational analysis, is a nontrivial exercise. Manual concatenation of phylogenetic matrices, for example, necessarily involves the time-consuming process of identifying and eliminating character redundancy (e.g., Gatesy et al. 2002; Gatesy and Springer 2004; Hill 2005). Ascertaining the presence or the absence of a morphological feature requires an additional effort to reason from text that may only incidentally describe an aspect of it. As a consequence, the ability of scientists to hand-assemble data across studies is severely hampered by the difficulty to compute on taxa and morphological data. Our work shows that computable phenotypes not only enable automatic consolidation of character states into nonredundant presence/absence assertions, but they enable inference of presence/absence generalizations. Our method makes data reuse by nonexperts not only more efficient, but also reduces the risk for error and expands the phenotypic and taxonomic coverage of the original data. In so doing, it can open new possibilities for data analysis, in particular if phenotypes are linked with genes and other data through their shared ontological context (Mabee et al. 2012).

### Inference Expands Data: Filling in the “Unknown Knowns”

Our results demonstrate that inference can play a profound role in supplementing the taxonomically sparse phenotype assertions across taxa (Fig. 4). We found that 76% of the variable characters in the synthetic supermatrix were made that way through inference, meaning that at least one of their two states (presence or absence) is based solely on inferred data. For an individual feature such as the skeleton of digitopodium (the collection of skeletal elements encompassing the digits, i.e., the metacarpals/tarsals and phalanges), the number of inferred assertions (7751 annotations for 718 taxa) is 38 times higher and spread over 7 times more taxa than direct assertions (201 annotations for 103 taxa). An individual taxon can have multiple sources of inference for an individual entity, depending on the number and nature of the annotated characters that relate to that taxon and entity. For example, *Acanthostega* has 5 directly asserted and 30 inferred sources with character states that entail presence or absence of “skeleton of digitopodium.”

At the most basic level, aggregation of and inference on phenotype data allows users to supplement large amounts of missing data computationally, without extensive manual literature research. As Hiller et al. (2012) show, simply knowing in which taxa a phenotypic trait is present or absent across a taxonomic range in which the trait underwent evolutionary change can enable entirely new insights into the developmental genetics of the trait. Although the overall goal of our method, filling in data that is not directly asserted, is similar to imputation, our method differs substantially from this technique. Regression-based imputation practices for finding the “invisible fraction” (Grafen 1988) use probabilistic models to reconstruct the “unknown knowns,” whereas our method bases its reconstructions on predefined logical axioms in the ontology. Imputation methods can be effective at reducing gaps in quantitative data sets (Nakagawa and Freckleton 2008, 2011; Swenson 2014); however, they are not applicable to qualitative matrix data. Our use of inference to extract unstated knowledge about the presence and the absence of traits allows reconstruction of missing values without resorting to statistical parameters that may change across phylogeny. Though we restrict ourselves to a simple set of relational rules for entities and their parts that are uniform across metazoans (i.e., the humerus, when present, is always *part_of* the forelimb), the results of the logical reasoning methods used here are very powerful.

The supermatrix technique is a total evidence approach in systematics (Kluge 1989), where different data sets and types are combined into a single “supermatrix” of unique taxa (Sanderson 1998; De Queiroz and Gatesy 2007). Inevitably, component data sets overlap, but incompletely, resulting in many taxa lacking data for many characters. In the realm of molecular data matrices, there have been two approaches to deal with the missing data problem: (i) leave all taxa separate and code the unavailable characters as missing; or (ii) reduce missing data by making composite taxa at a level for which monophyly is assumed *a priori*. The former of these may lead to loss of resolution but not necessarily misleading relationships (Kearney 2002; Kearney and Clark 2003; Wiens and Morrill 2011; Wiens and Tiu 2012). The latter, composite taxa, may lead to misleading phylogenetic results (Malia et al. 2003). Whether supermatrices are sequence or morphology based, they typically involve a lot of missing data. Molecular supermatrices may include over 70% missing data (De Queiroz and Gatesy 2007; Fabre et al. 2009; Hejnol et al. 2009; Hedtke et al. 2013), and a morphological supermatrix assembled by Ramírez et al. (2007) had 94% missing data. By comparison, missing data in the variable character-subset of the synthetic supermatrix we created amounted to 98.5% without applying inference, and applying logical inference reduced this fraction to 78.2%.

### Characteristics and Application of Isomorphic Synthetic Characters

A considerable fraction (nearly 60%) of the populated cells in the variable subset of the synthetic supermatrix are characters that are part of one of 93 isomorphic clusters. That is, they corresponded to entities whose presence/absence distributions are identical across taxa (Table 4 available as Supplementary Materials on Dryad at http://dx.doi.org/10.5061/dryad.rm907). That our matrix synthesis method generates isomorphic clusters is expected, because presence/absence reasoning uses axioms in the Uberon anatomy ontology about part–whole and developmental precursor relationships (Balhoff et al. 2014). This will necessarily result in clusters composed of an asserted entity and its containing (for presence) or contained (for absence) classes, and/or developmental precursors (for presence) or derivatives (for absence). For example, for a character of “femur bone,” a state value of present will induce the same state value for characters “hindlimb,” “limb,” “femur cartilage element,” “femur pre-cartilage condensation,” and so on (see Fig. 3). We found that 10% (9 of 93) of the isomorphic clusters were of this kind. Most clusters were composed of only inferred data, indicating that the underlying original character states did not directly assert presence or absence. Although some of these (21 clusters) could be identified as the consequence of logic equivalence chains for implied presence or absence (see “Results” section), the majority (63 clusters) resulted from various chains of inference from multiple entities with no obvious repeating patterns. For example, the taxonomic distributions of presence for “nail,” “dorsal skin of digit,” “distal limb integumentary appendage,” and “digit skin” are identical, even though the ontological presences of these entities are not inferred to be equivalent.

Whether and what value or impact these isomorphic clusters have will depend on the goals of the researcher using these data. Perhaps the broadest and most forward-looking applications for phenomic data involve understanding trait evolution in relation to other phenotypic traits, environmental factors, and aspects of development and related genomic features. For research questions such as these, the biological knowledge revealed by a cluster of correlated characters may have substantial value in supplementing the input data. For example, the presence of developmental precursors (femur cartilage, femur condensations) entailed by an entity's presence (e.g., the femur bone) may involve different genes and networks relevant to a developmental biologist. One can also envision research questions where the inferred data hold value that the asserted data do not. For example, the inferred presence and absence of “hindlimbs” would be valuable for studies examining correlations of habitat and locomotion, whereas the phenotype assertions that entailed them may not be directly related to locomotion (e.g., toenail color). Further, the knowledge structure that is laid out in sets of isomorphic characters may be of benefit as approaches to computationally dissecting out the expression of genes and their regulators (Hiller et al. 2012) are scaled up.

For researchers interested in using these matrices for phylogenetic reconstruction, caution must be exercised. The character dependency implied by a significant fraction of isomorphic (even if otherwise variable) characters suggests that synthetic matrices, at least in the form of presence/absence-only data, are not immediately suitable for phylogenetic reconstruction. As discussed above, observed isomorphism does not necessarily imply logical equivalence, and hence whether characters should be merged or not due to putative dependency would need to be carefully examined for each case. For example, “nail” and “dorsal skin of digit,” though isomorphic in their taxonomic distribution in this data set, have different developmental bases, and can thus be argued to not be dependent.

A related issue is the potential for overweighting of groups of characters connected by inference chains. For example, the absence of a structure with many parts will necessarily result in all the parts inferred as absent, and the presence of a structure will necessarily cause recursively all its containing structures to be inferred present. The impact of this, and whether absence-driven or presence-driven overweighting of characters predominates, is beyond the scope of this study. Importantly, character overweighting already exists in the literature, and must thus be taken into account when composing supermatrices. In contrast to other approaches, our method allows precisely tracing back the inference chains to identify the sources and magnitude of overweighting.

More generally, the degree to which phylogeny can be recovered from binary presence/absence data alone has, to our knowledge, not been investigated. Certainly, presence/absence data are common in morphological data sets; Sereno (2009) gives a figure of 25%. However, the phylogenetic resolution attained in these studies require variation in other qualities (size, shape, texture, color, etc.). The ontological methods used here reduce data from these other qualities to presence/absence, thus changing the phylogenetic level at which the information is relevant. For example, if variation in vertebral shape across a set of taxa is reduced to the inference that vertebrae are present in these same taxa, it no longer contains information to resolve them. However, the presence of vertebrae is informative for resolving taxa at a higher level (i.e., as members of the clade Vertebrata). Though this issue will require further examination, it is likely that the inferred presence/absence data will only support the monophyly of more inclusive clades than the original assertions.

### Distribution of Data across Taxa and Anatomical Regions

The paired appendages are ostensibly one of the most intensely studied aspects of anatomy in vertebrates, and yet quantifying the data available for them has not previously been possible. The methods presented here readily enable this, including visualizing how our knowledge of morphology, whether expressly stated or implied, is distributed over taxonomic and anatomical space (Fig. 5). This can then be used to pinpoint the taxonomic groups and the parts of the anatomy for which data are sparse or lacking, allowing potential reasons and remedies to be considered. One should note in this context that availability and lack of data for an anatomical feature in a taxonomic group should not be expected to coincide with presence and absence, respectively, of the feature in said group. Figure 5 illustrates this, for example, for digits in the lungfish *Dipterus*. Lungfishes do not have digits, yet due to assertions about their absence in this taxon (Zhu et al. 1999; Swartz 2012) data about digits in lungfishes are available.

For the matrix we synthesized for the evolution of vertebrate fin/limb morphology, the gaps in the data may be primarily attributable to the following two factors. One, most taxa studied in the fin-to-limb transition are fossils and thus restricted to a few, often partial, specimens. These taxa may also be unscorable for certain entities due to primitive absence (e.g., the ilium, ischium, and pubis of the pelvis are not present in basal nontetrapod taxa). Two, the taxa and anatomical elements used for study are unequally sampled. As is evident in Figure 5, there are much less data about the hindlimb relative to the forelimb in basal tetrapods, which cannot be explained by hindlimb specimens being unavailable or far less preserved in the fossil record for the respective taxa than their forelimbs. Hence, other explanations are needed. Perhaps the differences could be due to more variability, and therefore more character data, in the forelimb than the hindlimb during the fin-to-limb transition, which would be consistent with the “front wheel drive” hypothesis, which posits that the fin-to-limb transition was driven primarily by changes in the forelimb (Shubin et al. 2014). Alternatively, the difference could be a result of sampling bias caused by the larger size of the ancestral forefin and the interconnectedness of the girdle skeleton with dermal skull elements.

Regardless of what is really behind the difference, our results illustrate how the ability to visualize the uneven distribution of knowledge can reveal far more than simply the existence of bias. Gaps in morphological knowledge, such as regarding the phenotypic evolution of the hindlimb, can present major challenges for understanding the origins and evolution of novel features (Shubin et al. 2014), and the ability to synthesize knowledge on a large scale can focus future studies on filling in gaps.

### Quantification of Taxon Scoring

As a consequence of the obstacles to integrating morphological character data, it has been nearly impossible to assess quantitatively the differential sampling of taxa and anatomy across studies. This, too, is readily enabled by the methods described here. As an example, we examined how frequently individual taxa had been scored for fin and limb phenotypes in the generated synthetic supermatrix. Because of the logistic efforts necessarily involved in morphological data collection (specimen preparation, museum collection visits, etc.), the taxonomic sample of species that an investigator can examine is limited, and some taxa are more readily available for study than others. In our data set, 70% of the taxa in the synthetic supermatrix were connected to only a single publication record (Table 5 available as Supplementary Materials on Dryad at http://dx.doi.org/10.5061/dryad.rm907). For taxa having more than one source publication, the proportions drop rapidly: 12% and 7% are found in two and three publications, respectively, and fewer than 2% of the taxa are scored in seven or more publications. A single taxon, *Acanthostega*, a well-preserved exemplar taxon in the fin-to-limb transition, holds the maximum number of 16.

However, this distribution, and in particular the high proportion of single-source publication taxa, is unlikely to be representative of the vertebrate comparative fin/limb morphology literature as a whole. This is because the publications we chose for phenotype annotation treat mostly nonoverlapping sections of the vertebrate phylogeny, and thus a high fraction of taxa with a single publication source is a consequence of our experimental design. If we consider only the data for basal sarcopterygians relating to the fin-to-limb transition (Table 1 available as Supplementary Materials on Dryad at http://dx.doi.org/10.5061/dryad.rm907), the proportion of taxa with only a single publication source drops to 34%. However, when considering the fraction of taxa whose morphological features have been scored by only a single research group, this figure is likely an underestimate. Some of the publications in this subset of the supermatrix share co-authors, and many characters are recycled. A more thorough study of independence and depth of evidence across the data set was beyond the scope of this study, but our results illustrate how our methods would readily enable such an analysis.

### Conflicting Data Revealed

When authors reuse characters from previous works, encountering, and resolving coding conflicts is an important part of the process to ensure phylogenetic relationships are as accurate as possible (Harris et al. 2007). Character conflicts are often difficult to spot by hand, yet the protocols authors follow for identifying, adjudicating, and resolving conflicts are rarely reported beyond a high-level summary. The presented supermatrix synthesis approach immediately reveals conflicting phenotypes, here in the form of an anatomical feature having state values of both present and absent (0/1) for the same taxon. We found 774 such cells (0.5%) among the 146,451 populated cells, excluding directly asserted polymorphisms, which we defined as those that trace back to direct assertions of both states in the same source matrix (see “Methods” section). How this level of character conflict compares with what has been observed previously is difficult to assess, because in previous studies in which morphological matrices have been concatenated manually (see O'Leary et al. 2013; Sigurdsen and Green 2011), the resolution of conflicts is not reported in a quantitative manner. However, in a consensus morphological matrix for turtles, Harris et al. (2007) reported <2% cells with conflict (out of 4872 total cells), which is similar in magnitude to our finding.

One of the major advantages of the synthesis approach we present is not only that the extent of character conflict can be quantified quickly, but also that detailed reports about the provenance of all conflicting data can be generated automatically. This greatly aids review, and where possible, resolution of these data by experts. There are a number of different causes for an observed conflict, only some of which are correctable errors; determining the cause for a given character conflict requires careful examination. A trivial case stems from the fact that most matrices are not yet archived in digital repositories (Stoltzfus et al. 2012; Drew et al. 2013), and errors could be a result of their required manual digitization. These will be reduced by the increasing push for digital archival of matrices upon publication. More substantive conflicts, however, result from differing author assertions that may stem from observations of different (and differing) specimens or different interpretations of the same material. Additionally, the conceptualization of the character by the original author, and the terminology used for its description, may have consequences beyond the confines of the original state structure when annotated with ontology terms that have logical implications, leading to conflicting results. A discussion of conflicts in relation to their bases in assertion and/or inference follows, with examples from the data set we generated. It is worth noting that for conflicts due to correctable errors, our fully computational approach to matrix synthesis has the advantage that once the errors are addressed in the KB, the corresponding conflicts are eliminated from any supermatrix subsequently generated from it.

#### Conflicts between Asserted Character States

The conflicts that are most readily traced to their cause are those between authors who differently assert the presence and the absence of an anatomical structure. These comprise a relatively small proportion of the conflicts (17%). Some of these discrepancies arise as new observations are made, for example, from new specimens that reveal formerly poorly known anatomy. For example, Zhu et al. (1999) scored the humerus of *Strepsodus*, a rhizodontid fish, for the presence of distinct supinator and deltoid processes. Based on new fossil material for rhizodontids, the humerus morphology was re-evaluated by Jeffery (2001) who concluded that in *Strepsodus* and other rhizodontids distinct supinator and deltoid processes are absent, thus generating the conflict observed in our data set.

Sometimes, the basis of conflict between original author assertions is not as readily traceable. For example, in the fossil literature it is not uncommon that not all of the specimens examined in relation to each operational taxonomic unit (OTU) are reported. Even when specimens are listed comprehensively, the reasons for conflicts are sometimes difficult or even impossible to deduce from the published literature alone. For example, Ruta et al. (2003) state that accessory foramina (passages for blood vessels) are absent in the humerus of the fossil amphibian *Sauropleura*, but later Ruta (2011) scored these foramina as “present.” As it does not appear from his documentation that different specimens were examined, this leaves re-examining the specimens or communicating with the authors as the only resort to resolving the conflict. Such differences in scoring are a challenge for both manual and machine concatenation of these data, but they are to be expected, as authors not only have access to different materials over time, but will also sometimes vary in their interpretation of structures. The presented matrix synthesis method cannot reduce or eliminate them, but it is able to readily pinpoint candidates for investigation, including by way of computationally (and thus automatically) generated reports.

#### Conflicts between Asserted and Inferred Character States

The most frequent conflicts (73%) occur between asserted and inferred data. These are arguably much less obvious from manual analysis than the detection of conflicting assertions. An example comes from a recent large-scale examination of tetrapod limb evolution, focused on the transitional fossil *Tiktaalik roseae*, which is described as having a “poorly developed” scapula blade (Ruta 2011). This assertion results in its inferred presence in the synthetic supermatrix (Fig. 1). The scapula blade, however, is directly asserted to be absent in *Tiktaalik roseae* by Swartz (2012) and Clack et al. (2012). Regardless of what is at the root of this conflict (different specimens, different interpretations of morphology, polymorphism, etc.), the value of our method is that it makes the discrepancies in the literature evident.

#### Conflicts between Inferred Character States

The fewest conflicts (10%) are generated between data based on inference alone. For instance in the frog *Bombina variegata*, the ilial protuberance is inferred absent based on the assertion that the ilial shaft is absent (Fabrezi 2006), of which the ilial protuberance is a part. The presence of the ilial protuberance is inferred from two assertions regarding its shape, that is, “not knobbed distally” and “broad and low rounded” (Cannatella 1985), thus generating a conflict. Identifying the condition(s) in this species is beyond the scope of this article, but it would likely require the user to analyze the supporting specimens from the original sources. Again, the value of our method is that it reveals the conflicts, here from inference alone, which particularly in this case would be difficult to ascertain manually.

#### Conflicts from Author Character Structure and Scoring

Some “false” conflicts resulted from the idiosyncratic character construction and scoring practices by authors, and also limitations of the KB. For example, a conflict is automatically generated when an author creates a character state that is a disjunction of absence and one or more other qualities that entail presence. For example, the character, “ectepicondyle” with the state: “low, indistinct or absent” (Laurin and Reisz 1997) is intended to reflect the variability present across the taxa. Yet this wording does not allow the reader to differentiate whether this represents polymorphism within species (i.e., different states in different individuals of a single species), or whether the set of species to which the description applies has multiple states (i.e., one state in one species, a different one in another). In this case, because “low” implies the presence of an ectepicondyle, it is automatically shown as in conflict with the same authors' assertion of absence. This illustrates how ambiguity in how an author constructs character states can limit or even preclude the utility of their data in other contexts.

Another source of error stems from character constructions that involve phenotypes of anatomical elements that are more complex than simple presence/absence, but are applied to taxa to which strictly speaking they do not apply. For example, the frog *Rhinophrynus dorsalis* is asserted to lack a sternum (Cannatella 1985). Yet in the same study the epicoracoid bone is scored in this taxon as “not fused to sternum,” from which a machine reasoner, and arguably also a human reader, would infer that a sternum is present. If an author scored this as “not applicable” in the original matrix, the logical error (inferred presence) would be avoided.

Inferences depend on the data asserted by the author. For example, the loss of digit I (thumb) in amniotes might be denoted as “digit I absent” or “four digits present” (assuming the plesiomorphic presence of five). However, if the identity of the absent digit is not specified by the author, the annotation and reasoning methods described will not be able to infer it. Thus we strongly urge authors, when dealing with the presence/absence of serially homologous structures in particular, to be explicit regarding the identity of the entity in question.

Finally, inattention to the semantics of anatomical terminology can lead to incorrect and conflicting assertions. For example, although there is clearly a deep homology across Sarcopterygii between distal fin (paired fin radials) and distal limb (digits) skeletal elements (Johanson et al. 2007), they are generally considered distinct. Yet in the synthetic matrix, some limbed tetrapods are inferred to possess both radials and digits. This inference was generated from several limbed, and potentially terrestrial tetrapod taxa such as *Acanthostega*, *Dendrerpeton*, and *Silvanerpeton*, scored as possessing “jointed radials” (Swartz 2012). Thus the presence of radials is inferred for these taxa, while simultaneously digits were directly asserted for them (Ruta 2011). Perhaps Swartz (2012) used “radial” to encompass all acropodial elements because there is simply not a more encompassing anatomical term that applies to these distal skeletal structures across the taxonomic breadth of vertebrates. This use of “radial,” however, conflicts with its general usage in the literature (Ruta 2011) as well as genetic data concerning the distinctness of digits (Davis 2013; Woltering et al. 2014). Referencing and applying a standardized vocabulary in character descriptions would resolve this type of conflict (Seltmann et al. 2012).

As described above, author-generated conflicts pose a problem to the effort of automatic integration of these manually annotated character data. Because they are idiosyncratic and difficult to detect until integration, there is little possibility to create filters that automatically correct for these types of errors. We suggest it is better to work to amend character construction practices, and working toward a future in which characters are constructed in computable form *a priori,* than trying to address them *post hoc*. This will likely become even more important as text markup of phenotypes and other concepts is automated, leaving little margin for human curator correction of inconsistencies.

### Improved Annotation and Curation Standards

The annotation practices that guided the EQ assignments to the character data in Phenoscape were designed to capture the rich anatomical detail and differences among taxa, as described by taxonomic experts. Combining and reasoning across the annotations in this study, however, cast these data in different relief, in some cases revealing conflicts that were the result of inappropriate annotation of author statements. Resolving these, and generalizing the issues where possible, enabled us to improve and expand the anatomy and quality ontologies, the annotations, and the phenotype curation guidelines (http://phenoscape.org/wiki/Guide_to_Character_Annotation). For instance, it appeared that data from a single paper conflicted in whether or not the fish *Onychodus* possessed a postcleithrum (Cloutier and Arratia 2004). Investigation revealed that the authors directly asserted the absence of this bone; an inferred but incorrect presence resulted from a mistake in annotating “presence of a postcleithral scale” as “‘dermal scale’ and *part_of* some postcleithrum, present.” The postcleithral scale, however, is a separate type of scale and not a part of the postcleithrum bone. In this case, we added a new entity “postcleithral scale” to the Uberon ontology as a type of “scale,” the feature was re-annotated, and the conflict thus removed.

## Future Directions

The approach and methods demonstrated here to compute synthetic presence/absence supermatrices are applicable to any taxonomic and phenotypic slice across the tree of life, provided these data are semantically annotated. Scaling up annotation to this level, however, will require significant effort, including the development of semiautomated methods for marking up free-text descriptions (e.g., Cui 2012; Arighi et al. 2013; Dahdul et al. 2015); provisioning of community phenotype ontologies to accommodate the diversity of taxa and evolved anatomies and qualities (Gkoutos et al. 2005; Haendel et al. 2014); and faster and more efficient methods for reasoning across these substantially larger data in knowledgebases. Another challenge lies in developing methods to aggregate “non” presence/absence phenotypes, that is, those features varying in qualities such as size, shape, color, texture, etc., into a matrix format, which will require sophisticated algorithms for automating consolidation of synthetic character states. Additionally, new methods are required to integrate taxonomically-heterogeneous supermatrix data with user-specified trees. Because the phenotype data are asserted at multiple taxonomic levels (i.e., to species, genera, families, etc.), current methods for their optimization and visualization along a tree are limited. The power of leveraging ontology-based reasoning to propagate anatomical knowledge over phylogenetic trees has been recently demonstrated (Ramírez and Michalik 2014). Using this approach, in conjunction with the synthetic matrices demonstrated here, indicates a necessary/critical role for inference in understanding patterns of phenotypic evolution across large data and trees.

## Conclusions

The phenotypic features that characterize and define evolutionary groups are currently scattered across the dispersed literature of comparative biology, often in character-by-taxon matrices for small sets of taxa. The difficult and time-consuming manual aggregation of these data reduces their reuse. Here we demonstrate that when phenotypes are ontology-annotated, their presence and absence can be automatically integrated into synthetic character matrices. We found that inference plays a profound role in supplementing the taxonomically sparse phenotype assertions across taxa, in our case reducing the missing data in the variable character-subset from 98.5% to 78.2%. Moreover, 76% of the variable characters were in fact made variable through the addition of inferred presence/absence states. Equally important, this automated method results in immediate isolation of character conflicts and detailed reports about their provenance. This capability, if available broadly, will greatly aid experts in data review, and where possible, conflict resolution. Finally, machine reasoning enables quantification and new visualizations of the data, as demonstrated here, allowing the identification of character space that is undersampled across the fin-to-limb transition.

## Supplementary Material

Data available from the Dryad Digital Repository: http://dx.doi.org/10.5061/dryad.rm907.
